# Root hydraulic conductivity and adjustments in stomatal conductance: hydraulic strategy in response to salt stress in a halotolerant species

**DOI:** 10.1093/aobpla/plv136

**Published:** 2015-11-24

**Authors:** Victoria Vitali, Jorge Bellati, Gabriela Soto, Nicolás D. Ayub, Gabriela Amodeo

**Affiliations:** 1 Departamento de Biodiversidad y Biología Experimental, Facultad de Ciencias Exactas y Naturales, Instituto de Biodiversidad y Biología Experimental, Universidad de Buenos Aires and Consejo Nacional de Investigaciones Científicas y Técnicas, C1428EGA Buenos Aires, Argentina; 2 Instituto de Genética “Ewald A. Favret”, CICVyA, INTA-Castelar and Consejo Nacional de Investigaciones Científicas y Técnicas, 1686 Buenos Aires, Argentina

**Keywords:** Aquaporins, *Beta vulgaris*, root hydraulic conductivity, salt stress, soil–plant–atmosphere continuum, stomatal conductance, suberization, water relations

## Abstract

Recent advances at the molecular level are introducing a new scenario that needs to be integrated into the analysis of plant hydraulic properties. Although it is not yet clear to what extent this scenario alters the current proposal for the hydraulic circuit models, it introduces new insights when studying plants that are able to easily overcome water restrictions. In this context, our aim was to explore water adjustments in a halotolerant model (*Beta vulgaris*) by studying the coordination between the root in terms of root hydraulic conductivity (*L*_pr_) and the shoot as reflected in the stomatal conductance (*g*_s_). The root water pathways were also analysed in terms of root suberization (apoplastic barrier) and aquaporin transcript levels (cell-to-cell pathway). *Beta vulgaris* showed the ability to rapidly lose (4 h) and gain (24 h) turgor when submitted to salt stress (200 mM). The reduction profile observed in *L*_pr_ and *g*_s_ was consistent with a coupled process. The tuning of the root water flow involved small variations in the studied aquaporin's transcripts before anatomical modifications occurred. Exploring *L*_pr_ enhancement after halting the stress contributed to show not only a different profile in restoring *L*_pr_ but also the capacity to uncouple *L*_pr_ from *g*_s_. *Beta vulgaris* root plays a key role and can anticipate water loss before the aerial water status is affected.

## Abstract (precis)

In plants, the total hydraulic resistance adjustment is a crucial trait in tolerance. We explored the link between root and shoot by means of simultaneously characterizing root hydraulic conductivity and stomatal conductance in a halotolerant model (*Beta vulgaris*). Under salt stress, *B. vulgaris* triggers a hydraulic strategy tuning the root and shoot in a coupled process. After halting the stress, the profile of root efficiency at transporting water differed from that of shoot adjustment, underlining the relevance of the root for the overall hydraulic response: root capability allows recovery of water flow before the aerial water demand is restored.

## Introduction

Water flow through plants has been described as a passive mechanism (diffusion and bulk flow) based on the analogy with Ohm's law (Van den Honert 1948). The movement of water along a hydraulic circuit with resistances (*R*, m^−3^ s MPa) to the water flow at the root, shoot and canopy levels is known as the soil–plant–atmosphere continuum or SPAC (Tardieu and Davies 1993; Suku *et al.* 2014). Given a water potential gradient (ΔΨ, MPa), an increase or decrease in the water flow (*J*_v_, m^−3^ s^−1^) will reflect a change in the hydraulic conductance (*L*_o_, m^3^ s^−1^ MPa^−1^) along the plant's hydraulic circuit. In this model, the daytime transpiration demand of the aerial part of the plant—modulated by stomatal conductance (*g*_s_, mmol m^−2^ s^−1^)—is the main contributor to the driving force that ensures water entry through the roots (Sack and Holbrook 2006).

Thus, hydraulic integration can be considered to be a trait with important implications for plant structure and function (Schenk *et al.* 2008). In the last decade, considerable attention has been given to discover how root hydraulic properties affect the overall water uptake. Despite analysing changes in the absorbing surface area or modifications in the driving force, a new approach is provided that considers the intrinsic water uptake properties of the root (hydraulic conductivity, *L*_pr_) as a key component of the capacity to transport water per unit surface and per driving force (Steudle and Peterson 1998; Steudle 2000; Tyree 2003). The discovery of aquaporins has contributed to a reconsideration of the paradigm of the membrane transport capacity in terms of water and/or certain solutes or gases (Maurel 1997; Javot and Maurel 2002; Tyerman *et al.* 2002; Hachez and Chaumont 2010; Alleva *et al.* 2012; Chaumont and Tyerman 2014). According to the ‘composite transport model’ (Steudle 2000), the magnitude of the osmotic and hydrostatic forces will determine which path is the primary contributor to water flow: the apoplastic pathway (with low resistance) and/or the cell-to-cell pathway (i.e. symplastic plus transcellular, with high resistance) (Steudle and Peterson 1998; Suku *et al.* 2014). However, it is not only a question of how limiting the radial water flow could be but also to what extent these two pathways can be modified to rapidly adjust the *L*_pr_. Recent findings emphasize that aquaporins might substantially contribute to water uptake (e.g. barley: Knipfer and Fricke 2011; soybean: Vandeleur *et al.* 2014). Evidence for the contribution of the radial water flow has been identified by applying hydrostatic pressure to the root medium (Boursiac *et al.* 2005; Hachez *et al.* 2012; Vandeleur *et al.* 2014) or by dissecting the hydrostatic and osmotic gradients in the entire plant (Fritz *et al.* 2010; Fricke *et al.* 2013; Gambetta *et al.* 2013).

The impact of the radial water flow on the hydraulic circuit could be analysed by studying the response of plants in conditions where the hydraulic driving force limits water absorption. For instance, salt stress is a condition in which both the excessive Na^+^ in the soil environment and the water deficit act as linked factors that severely affect the plant growth rate. High salt concentration reduces soil water potential and not only makes water absorption harder for the roots but also introduces toxicity through a gradual accumulation of ions in the plant tissues (Munns and Tester 2008). Thus, the fine regulation between the ion redistribution and the water flow pathways is crucial in the tolerance response. The relevance of membrane pathways involved in ion redistribution—particularly between Na^+^ and K^+^—has been well described (Niu *et al.* 1995; Peng *et al.* 2004; Karley and White 2009; Shabala *et al.* 2010; Gajdanowicz *et al.* 2011). It is still necessary to understand how water pathway resistances (or conductances) contribute to improve plant salt tolerance.

*Beta vulgaris*—a member of the Chenopodiaceaea family—is considered a halotolerant (Clarke *et al.* 1993) or moderately salt-tolerant glycophyte (Bartels and Sunkar 2005; Bartels and Dinakar 2013). This behaviour among beet subspecies is related to their versatile ability to accomplish a rapid osmotic adjustment by regulating their ion and water uptake (Daoud *et al.* 2008). In these plants, the decrease in the water potential imposed by salinity is overcome by osmotic regulatory mechanisms, and the plants gain the capacity to take up water from the saline medium and maintain their turgor. An isolated enriched fraction of *B. vulgaris* plasma membrane shows very high water permeability (*P*_f_ = 542 μm s^−1^; Alleva *et al.* 2006) that favours a highly permeable cell-to-cell pathway. To date, three *B. vulgaris* plasma membrane intrinsic proteins (*Bv*PIP1;1, *Bv*PIP2;1 and *Bv*PIP2;2) have been described (Qi *et al.* 1995; Barone *et al.* 1997, 1998) and characterized in a heterologous system (Bellati *et al.* 2010; Jozefkowicz *et al.* 2013). Because the *B. vulgaris* genome was very recently announced (Dohm *et al.* 2014), transcriptome global sequencing (Mutasa-Göttgens *et al.* 2012) as well as expressed sequence tag libraries provide excellent sources for open reading frame identification for tissue and/or different growth conditions (http://compbio.dfci.harvard.edu). The latter sources are precise enough to provide confidence that, to date, the three identified *Bv*PIPs described in this work remain the consistently abundant and highly expressed ones (Skorupa-Kłaput *et al.* 2015).

In an environmental condition with low water availability in the soil, the root water pathways can combine anatomical/architectural changes with the adjustment of aquaporin contribution, which might finally be reflected in the *L*_pr_ (Hachez *et al.* 2006; Maurel *et al.* 2010; Chaumont and Tyerman 2014). In particular, plants under salt stress might decrease *L*_pr_ by means of different strategies, including (i) the modulation of aquaporin by post-transductional mechanisms (Boursiac *et al.* 2005, 2008; Luu *et al.* 2012) or by transcriptional changes (Jang *et al.* 2004; Mahdieh *et al.* 2008; Horie *et al.* 2011; Muries *et al.* 2011; Liu *et al.* 2012) and (ii) changes in the root architectural arrangement (Galvan-Ampudia and Testerink 2011; Horie *et al.* 2012) and/or anatomical changes (Bramley *et al.* 2009), including suberin deposition (Krishnamurthy *et al.* 2011; Sutka *et al.* 2011). Thus far, the above-mentioned mechanisms described in (i) are associated with faster and reversible responses (hours–days), while those described in (ii) are related to long and irreversible acclimation triggered days after the onset of the stress (Munns and Tester 2008; Horie *et al.* 2012).

The aim of this work was to explore how hydraulic adjustments improve the tolerance response in a halotolerant species by analysing *L*_pr_ and *g*_s_ changes. The dynamics of root water adjustment (including water pathways) was explored under two salt treatments (200 mM NaCl and 200 mM KCl). Sodium ion was replaced with K^+^ to provide a source of a different monovalent cation as an inorganic osmolyte (Rahnama *et al.* 2010). This experimental design (NaCl versus KCl) was introduced because the ion redistribution is different, i.e. Na^+^ linked to the apoplast versus K^+^ linked to the transcellular pathway (Tester and Davenport 2003; Shabala and Cuin 2008). These redistributions will affect not only the water fluxes but also the water pathways involved. Our working hypothesis is that changes in resistances (or conductances) should also be accomplished to rapidly adjust the plant hydraulics. Although ABA and signalling crosstalk have been extensively addressed in the literature (Finkelstein 2013; Geng *et al.* 2013; Mittler and Blumwald 2015), the contribution of our work is to analyse in detail the hydraulic continuum associated with tolerance by performing a biophysical study to quantify the water adjustments.

To achieve this goal, our experimental design (NaCl versus KCl salt treatment) included (i) exploration of the plant hydraulic dynamics analysing two conditions that reflect different root–shoot water status in *B. vulgaris* (loss of turgor and gain of turgor) after being submitted to salt treatments and (ii) exploration of the hydraulic adjustment capacity to recover after the salt treatment is halted, thus assessing the contribution of the water pathways. We analysed physiological parameters linked to the water adjustment capacity at the whole-plant level: water potential, *g*_s_ and *L*_pr_, together with the amount of *Bv*PIPs aquaporin's transcripts and root anatomical modifications. Our hypothesis is that the tolerance of *B. vulgaris* to salt stress may be explained in terms of a high capacity to perform hydraulic adjustments and that this capacity might quantitatively reflect root plasticity that functions as a rheostat in the SPAC (Maurel *et al.* 2010).

## Methods

### Characterization of a new state for *B. vulgaris* under salt stress

#### Plant growth and experimental design

*Beta vulgaris* was grown under controlled environmental conditions with a 16/8 h light/dark cycle in a 21 °C conditioned growth chamber (light intensity conditions were 148 ± 10 µmol m^−2^ s^−1^). Red beet seeds were germinated in plastic containers filled with sterilized sand and moistened with hydroponic culture: 1.25 mM KNO_3_, 0.75 mM MgSO_4_, 1.5 mM Ca(NO_3_)_2_, 0.5 mM KH_2_PO_4_, 50 μM FeEDTA, 50 μM H_3_BO_3_, 12 μM MnSO_4_, 0.70 μM CuSO_4_, 1 μM ZnSO_4_, 0.24 μM Na_2_MoO_4_ and 100 μM Na_2_SiO_3_ (Javot *et al.* 2003). Ten days after germination, the healthy seedlings were transplanted into aerated hydroponic culture containers. Distilled water was added on the 10th day to compensate for the losses by evapotranspiration. For all of the studied parameters, a nutrient solution was complemented or not with NaCl or KCl (200 mM) at 21 days after planting, i.e. when the first true leaf was completely mature. The treatments were always started at the beginning of the light cycle (9:00 AM), which was considered to be time 0 h. The subsequent harvest time(s), where any parameter was measured and/or samples taken, are in reference to this initial (*t* = 0 h) time. All treatments were applied in a completely randomized design. At least three to four independent biological replicates were used in each experiment. Data are expressed as the mean of three performed independent experiments. The final salt concentration was selected by analysing the plant's response to different NaCl treatments (50, 100, 250 and 500 mM) **[see Supporting Information—Fig. S1]**. Our strategy was to find a physiological condition where hydroponically grown plants were able to rapidly show a clear change in their phenotype (loss of turgor), followed by a gain of turgor after the onset of salt stress. This phenotype change was remarkable at 200 mM NaCl (Ψ_medium_ = −0.90 MPa).

#### Relative water content

The first true leaf was collected from different plants at different time intervals after treatment and employed to determine relative water content (RWC), as described by Turner (1981). The turgid weight was measured on the same leaves after immersing them for 24 h (until the final weight value was constant) in distilled water, and the oven-dry weight (DW) was obtained after drying them at 70 °C for 24 h (until the final weight value was constant).

#### Transpiration rate

The volume of water transpired per plant was measured gravimetrically. The plants were grown as follows: 1 day before the treatment was applied, each plant was moved to an individual plastic container, which was sealed to prevent evaporation. Every plant was weighed every hour between 9:00 AM and 5:00 PM during four consecutive days. In each plant, the slope of mass = *f*(time) was employed to calculate the average mass lost per hour per leaf area per day for all treatments (6–9 plants). In all cases, we determined the leaf area only on the fourth day, and this value was used to calculate the transpiration rate.

#### Relative growth rate of leaf area

The leaf blades (first true leaf) of the plants were photographed with a digital camera, and the leaf area was measured with image analysis software (Image J ver. 1.37; http://rsb.info.nih.gov/ij). The relative growth rate (RGR) was calculated with respect to the ratio of *A_i_* (leaf area in a given time) and *A*_o_ (leaf area at the beginning of experiment), and the results were expressed as the natural logarithm of the relative leaf area (*A_i_*/*A*_o_) as a function of time (from *t* = 0 h—onset of the salt treatment—up to 48 h). The slope of the curve estimates RGR.

#### Shoot–root ratio

To analyse the biomass distribution, the shoot–root ratio was determined from the fresh weight in each experimental condition (control, 200 mM NaCl, 200 mM KCl at 0, 4, 8, 24 and 48 h of the imposed treatment).

#### Apparent leaf water potential

The leaves of the treated or control plants were placed in a plastic bag covered with Parafilm^®^ foil prior to measurement in a Scholander pressure chamber (BioControl, Model 4, Argentina) to determine Ψ_leaf_ (Scholander *et al.* 1965). The measured leaf water potential in this work is referred to as the apparent leaf water potential $\left(\Psi_{\mathrm{leaf}}^{\prime }\right)$ because in species such as *B. vulgaris*—which shows halotolerant features—the osmotic potential (Ψ_osm_) of the xylem is not negligible (Boyer 1969; Kaplan and Gale 1974). It is, therefore, considered as an estimator of the water potential (Turner 1981).

#### Apparent turgor-pressure component

The pressure component of the water potential $\left(\Psi_{p}^{\prime }\right)$ in the leaf was calculated as $\Psi_{p}^{\prime }=\Psi_{\mathrm{leaf}}^{\prime }-\Psi_{\mathrm{osm}}.$ In another set of leaves, we determined the Ψ_osm_ following a freezing protocol as previously described (Mahdieh *et al.* 2008). The osmolality of each sample was measured in a vapour pressure osmometer (Vapro 5520, Wescor, USA) **[see Supporting Information—Table S1]**.

### Linking root hydraulic response to the overall SPAC

#### Stomatal conductance measurements

Stomatal conductance was measured with a steady-state porometer (SC-1, Decagon Devices, Pullman, WA, USA) on the first true leaf in each plant, a completely expanded mature one. To avoid time-consuming measurements, we first demonstrated that the measurement of one leaf was sufficient per plant, i.e. the *g*_s_ profile was similar between leaves in each plant during the day (data not shown).

#### Root hydraulic conductivity measurement

Measurements were performed as previously described (Javot *et al.* 2003; Boursiac *et al.* 2005). In these experiments, the entire root system of a freshly detopped plant was inserted into a 50-mL tube filled with the same nutrient solution bathing the intact plant, and the root was then placed inside the pressure chamber (BioControl, Model 2, Argentina). The hypocotyl was carefully connected to a glass capillary tube using a low-viscosity dental paste (A+ Silicone, Densell) and was then threaded through the metal lid of the chamber. We determined the exudated flow (*J*_v_) induced by the pressure. Briefly, the excised roots were subjected to three pressures in a stepwise manner: 0.3, 0.4 and 0.2 MPa. The exudated flow was constant in all time periods of measurement (5–10 min in each pressure). After measurements, the DW of the root was obtained. The *L*_pr_ of each individual root system (in mL mg^−1^ h^−1^ MPa^−1^) was calculated from the slope of a plot of flow (*J*_v_) versus pressure divided by the DW of the root system **[see Supporting Information—Graph S1]**. Diurnal effects were discarded measuring both properties during the day in control plants. The change in treated plants was statistically significant and independent of the time of the day.

### Exploring root adjustments in terms of water pathways

#### Root anatomy

As described by Sharp *et al.* (2004), roots were cut in an equivalent position with respect to both root meristem and whole root length to warranty identical ontogenetic state for all the treatments. Fresh roots were cut into pieces 10 mm in length and incubated in 0.3 % w/v Sudan IV (Sigma-Aldrich) (in ethanol 70 %, v/v) for 1 h (Sutka *et al.* 2011). The root fragments were then rinsed in distilled water and finely chopped using a razor blade. The samples were mounted on slides in glycerol and observed with a microscope (Zeiss Axioskop 2, Japan). We found a better pattern for the Sudan IV red-stained root with respect to autofluorescence in the free-hand cross-sections, and measurements of *L*_pr_ can be made in the same sample without fixing the material.

### Quantitative real-time polymerase chain reaction for aquaporin gene expression

The roots were carefully and quickly harvested, frozen in liquid nitrogen and stored at −70 °C. The total RNA was isolated from 70 to 80 mg of tissue using ‘RNeasy Plant Extraction kit’ with ‘Plant RNA Isolation Aid’ (Ambion, Austin, TX, USA) according to the manufacturer's recommendation, ending the isolation with a digestion with DNaseI. For each sample, 500 ng of total RNA were converted into cDNA using oligo(dT) and M-MLV reverse transcriptase (Promega, Madison, WI, USA) according to the manufacturer's recommendation.

The transcript expression of *Bv*PIP2;1, *Bv*PIP2;2, *Bv*PIP1;1, *Bv*UBIep and *Bv*GAPDH genes was studied by real-time polymerase chain reaction (RT-PCR). The primers were designed based on published sequences of the aquaporins found in *B. vulgaris* **[see Supporting Information—Table S2]**. The selection of *Bv*UBlep and *Bv*GAPDH as the housekeeping genes was based on genes reported in *B. vulgaris* and information available in the literature (Reid *et al.* 2006; Wan *et al.* 2010). The mRNA abundance of *Bv*GAPDH and *Bv*UBIep was not significantly different between the treatments (data not shown).

Quantitative PCR (qPCR) was performed with a MyiQ cycler (Bio-Rad) in a reaction volume of 25 µL containing 12.5 µL of IQ Sybr Green Super Mix (Bio-Rad), 320 nM primers and 5 µL of a 1/500 dilution of cDNA. The RT qPCR conditions comprised 1 cycle at 95 °C for 5 min and 34 cycles at 95 °C for 45 s, 60 °C for 30 s and 72 °C for 1 min. Amplification data were collected during the extension step (72 °C). The efficiency of the primer binding was determined by linear regression by plotting the cycle threshold value versus the log of the cDNA dilution (Soto *et al*. 2010). The absolute RNA amount for each gene was determined in every qPCR experiment. The relative gene expression was calculated as the ratio of the initial gene quantity to the initial mean quantity of the housekeeping genes (Soto *et al*. 2011). Quantitative PCR experiments were independently performed three times with comparable results. The three PCR reactions were carried out in duplicate. The transcript levels of the three studied aquaporins under salt treatments were compared with an osmotic treatment imposed by a non-charge and non-permeable solute [polyethylene glycol (PEG) 6000] at a concentration of 23 % (*p*/*v*), which induces a Ψ_medium_ of −0.90 MPa. The purpose was to contrast aquaporin transcripts between ion signals (NaCl and KCl) versus non-charged non-permeable osmolyte (data not shown).

### Restoring salt-treated plants to control medium: *L*_pr_ and *g*_s_ recovery profiles

To measure the *L*_pr_ and *g*_s_ recovery profiles, the plants were first submitted to a salt treatment (200 mM NaCl or KCl) for 4 or 24 h and then transferred to a control solution (considered now *t* = 0 h). Root hydraulic conductivity and *g*_s_ were then measured at different time intervals (0, 1 and 24 h) to characterize the plant's capacity to restore *L*_pr_ and *g*_s_ when the salt treatment was halted. As a control, we first determined the *L*_pr_ values immediately after changing the detopped roots to a control medium. This protocol was crucial to discard the flows that could be artefacts due to injury exacerbated with the salinity treatment. Root hydraulic conductivity values in control medium were not significantly different from those measured in the saline treatment **[see Supporting Information—Graph S2]**. All values shown are the average of three independent experiments. In each experiment, *g*_s_ was measured in one leaf per plant in three different plants and two or three roots were detopped for measuring *L*_pr_ under each condition.

### Statistical analysis

Statistical analysis was performed using software GraphPad Prism 5.00 for Windows, GraphPad Software, San Diego, CA, USA, www.graphpad.com. Differences were accepted as significant with at least *P* < 0.05 employing analysis of variance (ANOVA) and Bonferroni tests as indicated in the figure legends.

## Results

### Characterization of a new state for *B. vulgaris* under salt stress

**Figure 1.**
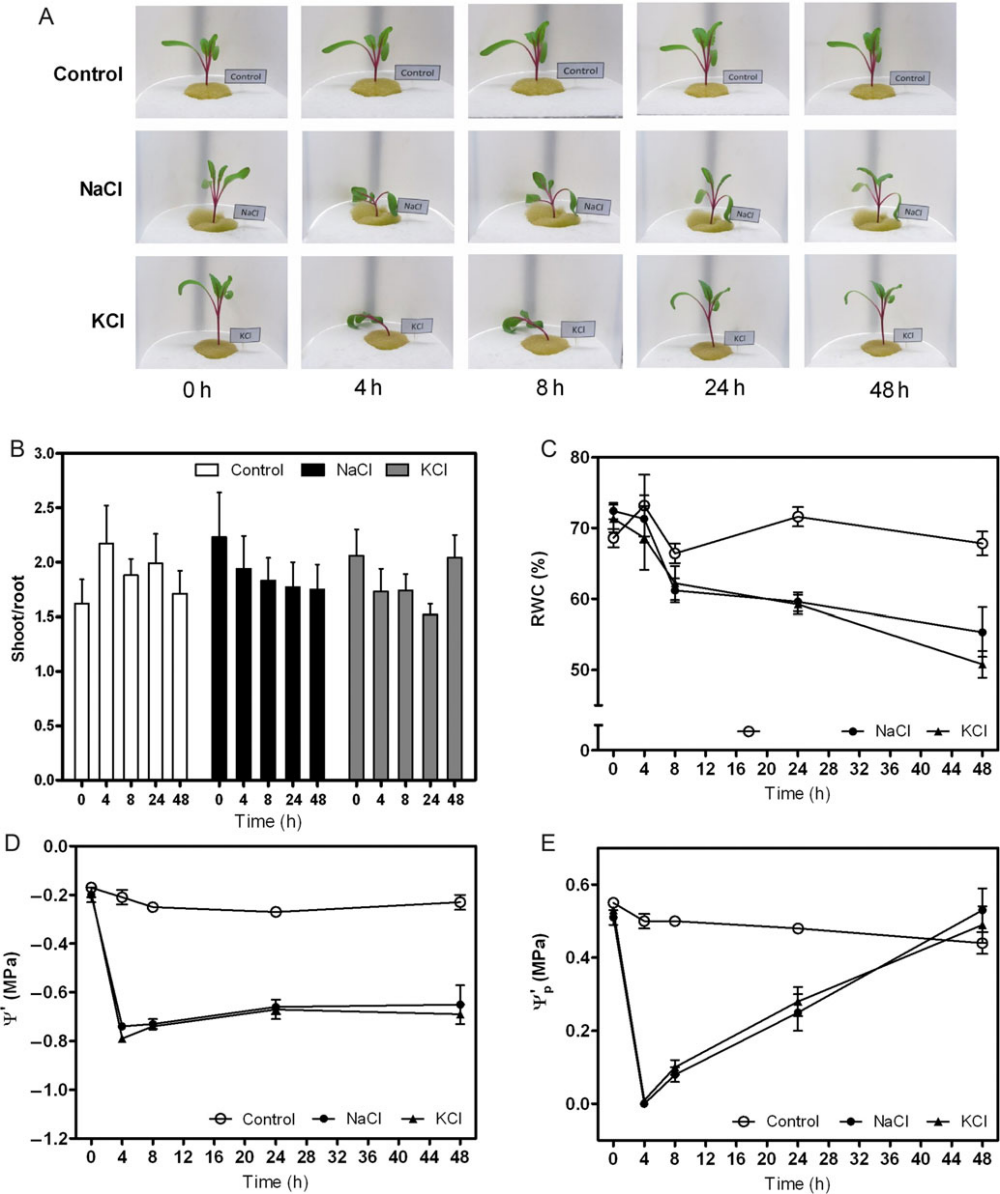
Effect of salinity treatments on *B. vulgaris* plants: control (Ψ_medium_ = −0.04 MPa); 200 mM NaCl (Ψ_medium_ = −0.90 MPa) or 200 mM KCl (Ψ_medium_ = −0.90 MPa). (A) Images of the same hydroponically grown plant taken in each condition at the indicated times after the onset of the treatment. (B) Shoot–root ratio values are given as bars representing mean ± SE of three independent experiments (*n* = 3). The biological replicates were five to six plants per treatment in each experiment. No differences were observed between treatments (*F*_(2,68)_ = 0.16, *P* = 0.8513). (C) Relative water content values are expressed as mean ± SE of three independent experiments (*n* = 3). The biological replicates were three plants per treatment in each experiment, and two leaves per plant were analysed as a duplicate. (D) The $\Psi_{\mathrm{leaf}}^{\prime }$ values (in MPa) measured at each indicated time are expressed as mean ± SE of three independent experiments. (E) The calculated apparent leaf pressure potential ($\Psi_{p}^{\prime }$ in MPa) is expressed as mean values ± SE.

Salt stress was achieved by the addition of 200 mM NaCl (Ψ_medium_ = −0.90 MPa). Under this condition, plants were able to rapidly show a clear change in their phenotype (loss of turgor) followed by a gain in turgor in <24 h (Fig. 1). The phenotype observed in plants exposed to 200 mM KCl was indistinguishable from the NaCl treatment (Fig. 1A), and no chlorosis symptoms were observed in the leaf. As expected, the tolerant phenotype shows a reduction in transpiration rate and in the leaf RGR in both salt treatments, although growth was not arrested **[see** **Supporting Information—Graph S3****]**. Moreover, during the whole experiment, the shoot–root ratio was not significantly modified, so the growth rate changes in the leaf were also translated to the root growth rate (Fig. 1B).

The water status of the hydroponically grown *B. vulgaris* plants was characterized. The RWC was reduced according to the phenotype observed (Fig. 1C). The $\Psi_{\mathrm{leaf}}^{\prime }$ was analysed at different time intervals (0, 4, 8, 24 and 48 h) for the control and treated plants. The mean $\Psi_{\mathrm{leaf}}^{\prime }$ in the control plants was −0.17 ± 0.02 MPa and remained constant during the whole experiment (Fig. 1D). When $\Psi_{\mathrm{leaf}}^{\prime }$ was measured after 4 h of the onset of the stress treatment, its mean value was significantly reduced in the NaCl condition (−0.20 ± 0.03 to −0.74 ± 0.01 MPa) and in the KCl condition (−0.19 ± 0.02 to −0.79 ± 0.01 MPa). The initial drop in $\Psi_{\mathrm{leaf}}^{\prime }$ is well correlated with plant turgor loss in both salt treatments (Fig. 1A). The $\Psi_{\mathrm{leaf}}^{\prime }$ remained at these low values up to 48 h although the turgid phenotype changed (Fig. 1). The leaf Ψ_osm_ remained constant in the control plants and showed a reduction in the plants submitted to stress after 24 h **[see Supporting Information—Table S1]**. The patterns of Ψ_p_ versus time were well correlated with the observed phenotype of loss and gain in turgor (Fig. 1A and E).

All of these parameters allowed us to define two distinguishable time intervals in terms of water adjustment during salt stress response, 4 h, where there is loss of turgor and 24 h, where there is gain of turgor. Our next step was to analyse these two conditions in terms of overall hydraulic adjustments.

### Linking root hydraulic response to the overall SPAC

**Figure 2.**
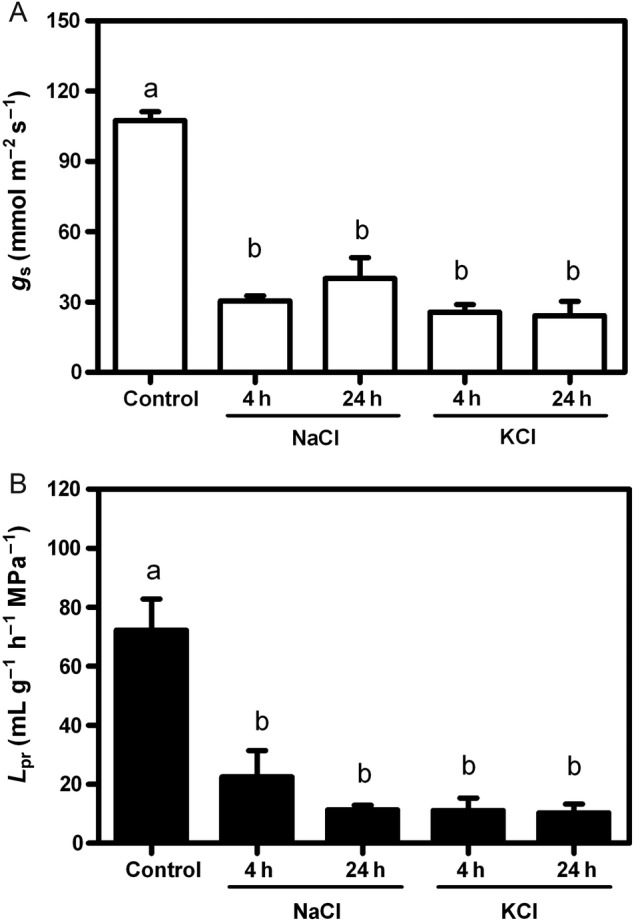
Integrating SPAC key points: *g*_s_ and *L*_pr_. (A) Stomatal conductance values (*g*_s_, in mmol m^−2^ s^−1^) are given as bars representing mean ± SE of three independent experiments (at least three plants per treatment). Different letters indicate statistical differences between treatments (*P* < 0.001; Bonferroni test). (B) Hydraulic conductivity values (*L*_pr_; in mL mg^−1^ h^−1^ MPa^−1^) are given as bars representing mean ± SE of three independent experiments (in each one, two to three individual root systems were measured). Different letters indicate statistical differences between treatments (*P* < 0.001; Bonferroni test).

As expected, the salt added to the medium triggered a rapid decrease in *g*_s_, which remained low even up to 24 h (Fig. 2A). In the control conditions, the plants showed mean *L*_pr_ values of 72.3 ± 21.1 mL g^−1^ h^−1^ MPa^−1^ (Fig. 2B). Both salt treatments induced a rapid and indistinguishable decrease in *L*_pr_. The *L*_pr_ inhibition was 80 % compared with the control condition after 4 h of treatment, and this low *L*_pr_ value was maintained up to 24 h of treatment. The *g*_s_ modifications are similar to the profile shown by *L*_pr_, suggesting that the change in the root water flow is coupled to *g*_s_.

### Exploring root adjustments in terms of water pathways

**Figure 3.**
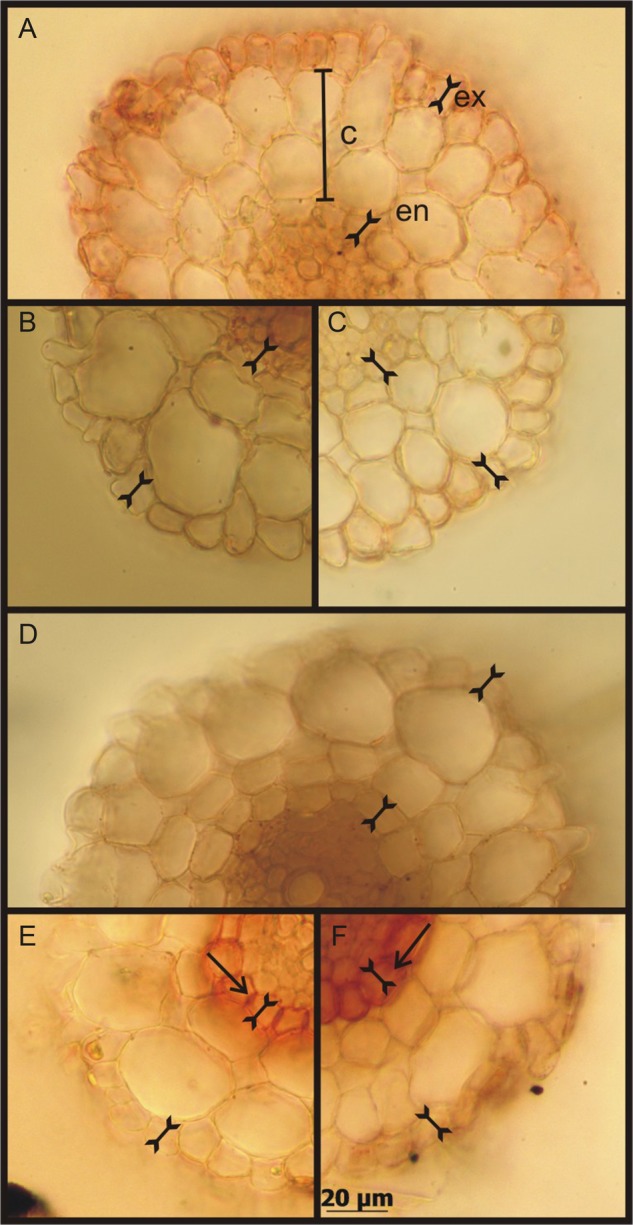
Photographs of *B. vulgaris* fresh root cross-sections stained with Sudan IV, the bar represents 20 µm. (A and D) Control root cuts indicating cortex (C), endodermis (en) and exodermis (ex). (B and C) Representative cuts of roots from plants treated for 4 h with 200 mM NaCl and 200 mM KCl, respectively. (E and F) Representative cuts of roots from plants treated for 24 h with 200 mM NaCl and 200 mM KCl, respectively. The arrows show suberization of endodermis. The images are one sample per condition of 10 independent experiments (*n* = 10).

The anatomical changes and the presence of aquaporins provide some insight into the putative involvement of the different water pathways at the root level for the two selected time intervals (4 and 24 h of treatment in 200 mM NaCl or 200 mM KCl). We, therefore, incubated root sections in the presence of Sudan IV in order to check suberization (Fig. 3). The plant roots challenged by either NaCl or KCl for a period of 4 h showed undetectable suberization changes of the endodermis and/or exodermis, as in the control plants. For longer exposures (24 h), the suberization of the endodermis increased independently of the ion treatment (Fig. 3E and F), whereas the control roots do not present enhanced intensity for Sudan IV. Similar results were observed when the autofluorescence of the cell wall was analysed (data not shown). These clear changes observed in the endodermis suberization were not observed in the exodermis. The exodermis suberization was very low and random, mostly attributable to higher thickness in the fresh cuts (Fig. 3A).

**Figure 4.**
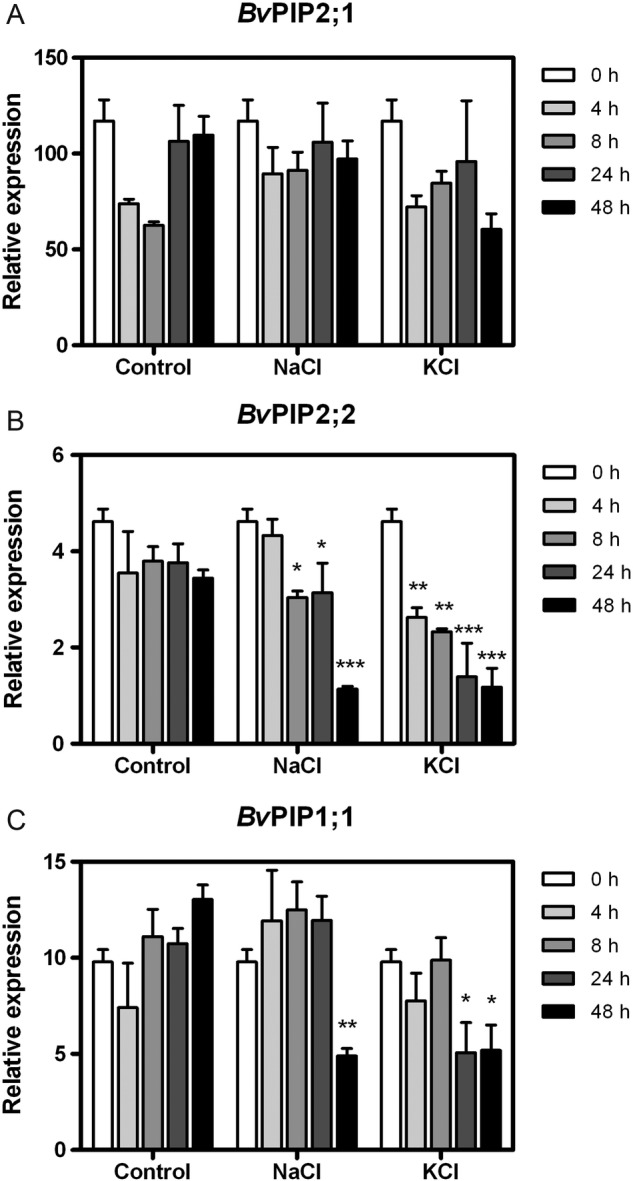
Relative gene expression of *Bv*PIP2;1 (A), *Bv*PIP2;2 (B) and *Bv*PIP1;1 (C) in roots from control, 200 mM NaCl or 200 mM KCl treatments. Data are given as bars representing mean values ± SE of three independent experiments (*n* = 3), and asterisks indicate statistical differences from initial condition (*t* = 0 h) for each treatment (*P < 0.05; **P < 0.01; ***P < 0.001; Bonferroni test) **[see Supporting Information—Tables S3–S5** for statistical analysis].

Quantitative RT-PCR analysis was performed to accurately determine the transcript levels of the PIP genes *Bv*PIP2;1, *Bv*PIP2;2 and *Bv*PIP1;1 in whole roots under salt stress (NaCl or KCl, Fig. 4) at different time intervals. *Bv*PIP2;1 might have a circadian behaviour as described in other PIPs (Takase *et al*. 2011; Caldeira *et al*. 2014). The studied aquaporins showed a subtle down-regulation profile, except the relative expression level of *Bv*PIP2;1, which did not decrease at all, independently of the treatment (Fig. 4A, **see Supporting** **Information—Tables S3–S5** for the statistical analyses). Interestingly, the profile of the *Bv*PIP2;2 and *Bv*PIP1;1 expressions for the NaCl stress condition did not show down-regulation at the same pace as observed in the KCl stress (Fig. 4B and C). The differences of the ion treatments became more evident for *Bv*PIP1;1, which showed down-regulation at 24 h when the plants were exposed to KCl, while the decrease became significant at 48 h under NaCl stress (Fig. 4C and **see Supporting Information—Tables S3–S5** for the statistical analyses).

To determine whether the lack of a strong down-regulation of these three aquaporins is only observed under salt treatment, the salt was replaced by a non-permeable and non-charged molecule as PEG. The plants submitted to Ψ_medium_ = −0.90 MPa generated with PEG triggered an expression decrease of 56–67 % in these aquaporins after 4 h of treatment, a strong transcript down-regulation compared with both salt treatment (NaCl versus PEG: *P* < 0.001, KCl versus PEG: *P* < 0.05, Bonferroni post-test).

### Restoring salt-treated plants to control medium: *L*_pr_ and *g*_s_ recovery

Our results confirm that the root water pathways are different in the two selected intervals (4 and 24 h) and that the three studied aquaporins are relatively stable upon salt treatments and only strongly down-regulated when a non-charge solute is imposed. However, the results do not allow us to completely dissociate the water pathways and ion redistribution (NaCl versus KCl). We decided to explore whether halting the salt treatment allows us to describe the shoot–root water dynamics through the analysis of *g*_s_ and *L*_pr_ recovery.

**Figure 5.**
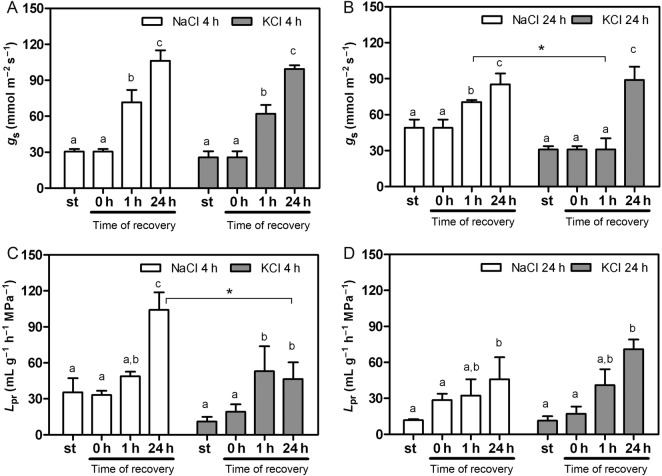
Recovery profile of *g*_s_ (A and B) and *L*_pr_ (C and D) after halting the salt treatment (st). The recovery profiles are separated for plants initially submitted to short salt treatments (*t* = 4 h, A and C) versus a longer interval period (*t* = 24 h, B and D). Data are expressed as mean values ± SE of three to four independent experiments (in each one, three to four plants were measured). Different letters indicate statistical differences between bars (*P* < 0.05; Bonferroni test), and an asterisk indicates differences between bars from different salt treatments (*P* < 0.001, Bonferroni test).

As shown in Fig. 5, the *g*_s_ recovery profile of the plants returned to the control medium reflected dependence of the time of the preceding salt treatment and dependence of the ion involved in the salt treatment. Thus, *g*_s_ recovered faster in the 4-h salt-treated plants than in the 24-h salt-treated plants (Fig. 5A and B). The 4-h salt-treated plants restored to the control solution for 1 h were able to increase *g*_s_ ≅ 50 % with respect to the *g*_s_ values before halting the treatment (Fig. 5A). In this analysed point (4 h of salt treatment before the halting), the recovery trend is independent of the involved cation (NaCl versus KCl; *F*_(1,22)_ = 3.06, *P* = 0.0940, two-way ANOVA). The plants subjected to NaCl or KCl for 24 h differed in their kinetic to increase *g*_s_ when they were restored to the control medium. In this condition (24 h of salt treatment), the ion involved in the salt stress significantly affects the recovery profile of *g*_s_ (ion accounts for 13.25 % of total variance, *F*_(1,16)_ = 12.99, *P* = 0.0024, two-way ANOVA). Even though *g*_s_ reached the same final value after 24 h of recovery in control solution, the recovery trend of *g*_s_ is much faster in NaCl-treated plants than in KCl-treated plants (Fig. 5B).

**Figure 6.**
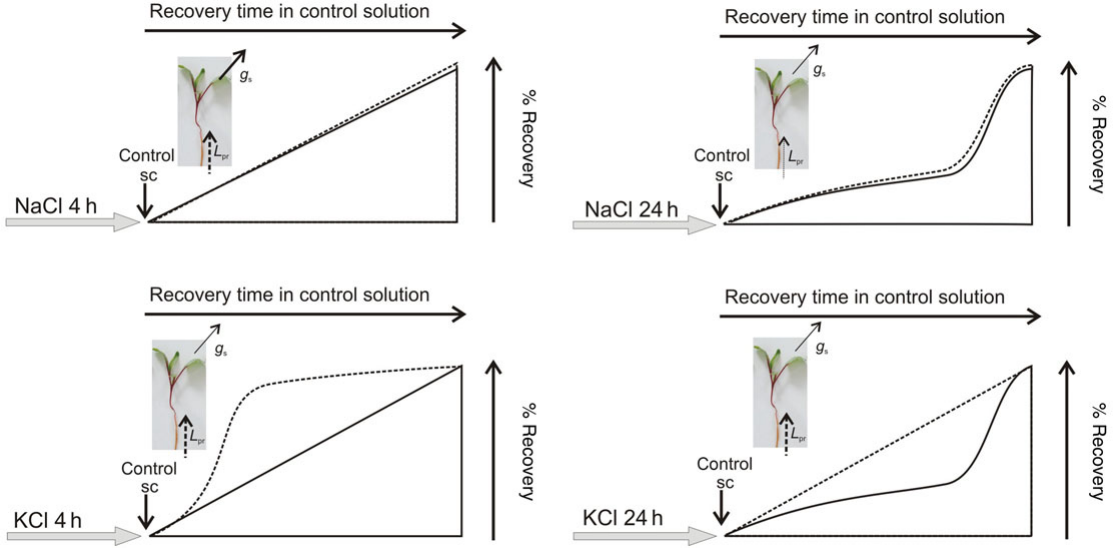
Schematic representation of recovery profiles of *L*_pr_ (dotted line) and *g*_s_ (continuous line) after stress treatments.

Conversely, the *L*_pr_ recovery profile of the plants restored to the control medium reflected a completely different strategy in terms of time dependence and ion dependence with respect to *g*_s_. Moreover, the recovery patterns of both hydraulic parameters (*g*_s_ and *L*_pr_) seem to be uncoupled although both presented a coupled reduction in salt treatment (Figs 5 and 6). In the first analysed condition (4 h of salt treatment), the recovery trend of *L*_pr_ is significantly affected by the ion involved before halting stress (Fig. 5C, *F*_(1,17)_ = 8.62, *P* = 0.0092). The 4-h KCl-treated plants turned into control solution showed a quick *L*_pr_ increment that remained unchanged for 24 h. In 4-h NaCl-treated plants, *L*_pr_ gradually rose to higher values (*P < 0.001; Bonferroni test). For the 24-h-treated plants, the trend of *L*_pr_ increment was independent of the salt treatment (*F*_(1,16)_ = 0.58, *P* = 0.4588, Fig. 5D). In the case of the NaCl-treated plants, *L*_pr_ recovery was affected by the extension of treatment (4 versus 24 h; Fig. 5C and D). The 4-h salt-treated plants presented a significantly higher *L*_pr_ value (*P* < 0.05; Bonferroni test) after 24 h of restoring the plants to the control solution. On the contrary, in the case of the KCl-treated plants, the *L*_pr_ recovery profile is independent of the extension of the treatment, i.e. 4 or 24 h (Fig. 5C and D). The 4-h-treated and 24-h-treated plants presented a similar *L*_pr_ value after 1 h of restoring the plants to the control solution, which was significantly different from the *L*_pr_ value observed under salt stress. Figure 6 illustrates *g*_s_ and *L*_pr_ recovery profile observed for the four conditions (NaCl or KCl; and/or the selected time points, 4 and 24 h).

## Discussion

Water homeostasis is linked to ion redistribution in plants as an important defence strategy against salt stress (Tester and Davenport 2003; Shabala and Cuin 2008; Shabala 2013; Flowers and Colmer 2015). *Beta vulgaris* showed great plasticity reflecting its ability to rapidly gain turgor due to osmotic adjustment, consistent with the maintenance of a low $\Psi_{\mathrm{leaf}}^{\prime }$ during the entire treatment (Fig. 1). Under our experimental conditions, low RWC values—even for plants gaining turgor after 24 h of salt treatment—might be associated with an underestimation of the RWC as a result of an osmotic adjustment **[see Supporting Information—Table S1]**. The gain of turgor under salt treatment requires solute synthesis and/or recirculation of cations, and this should also be reflected in the obtained RWC values (Weatherley 1950; Boyer *et al*. 2008). The aerial parts only modified 1 % of the water content (data not shown), even in the phenotype that lost turgor (4 h of salt treatment; Fig. 1), a trait consistent with an isohydric-like behaviour (Sade and Moshelion 2014). Thus, the overall strategy is successful for the adjustment of the water content. These data are supported by other studies performed using members of the Chenopodiaceae family under salt stress (Lindhauer *et al.* 1990; Ghoulam *et al*. 2002; Pakniyat and Armion 2007; Abbas *et al.* 2012), where Ψ_osm_ is the key element in turgor recovery. In our experimental design, the analyses were performed at 4 h (loss of turgor) and 24 h (gain of turgor) of salt treatment because these are two distinguishable transition states before a new water plant status is achieved.

The transpiration rate and *L*_pr_ have not always been reported as a coupled process. For instance, changes in shoot transpiration are not reflected by changes in *L*_pr_ in *Lotus japonicus* (Henzler *et al.* 1999), while in wheat, it was reported an important correlation between increasing *L*_pr_, the cortex cell hydraulic conductivity, transpiration and the root expression of aquaporins—*Ta*PIP1;2 and *Ta*PIP2;5 (Wang *et al.* 2013). Our results clearly showed that under salt treatment, there is a correlated decrease in *g*_s_ and root hydraulic properties (*L*_pr_) (Fig. 2), as both parameters presented an 80 % reduction compared with the control condition. The decrease in *g*_s_ (Fig. 2A) remained low even up to 24 h, which is consistent with the decrease in the leaf water potential values (Fig. 1D). This occurs for both NaCl and KCl treatments and is in agreement with observations performed in other species, such as wheat, that similarly decreased their *g*_s_ when exposed to either NaCl or KCl (Rahnama *et al*. 2010). The transition of the phenotypes—loss (4 h) and gain (24 h) of turgor—is not reflected in the two key water balance modulators (*L*_pr_ and *g*_s_) that remained coupled and similarly low. The hydraulic parameters only reflect a centred strategy of water loss avoidance.

Thus, it is necessary to explore how the root copes with water loss not only in terms of hydraulic properties but also in the analysis of the water pathways. The *L*_pr_ decrease (Fig. 2B) in our experimental set-up was consistent with other observations for different species (Martínez-Ballesta *et al.* 2003; Boursiac *et al.* 2005; Postaire *et al.* 2010; Muries *et al.* 2011). The roots showed a marked ability to adjust their *L*_pr_ during the first 4 h of treatment (our first hydraulic transition point) even before plants display any anatomical or morphological change (Fig. 3). It is consistent with faster responses that are usually present in the initial time lapse response to tolerance (Horie *et al*. 2012). In both 4 h salt treatments (NaCl and KCl), the suberization is indistinguishable from the control plants. The root apoplastic pathways were not modified, so the cell-to-cell pathway could be limiting (or maximizing) root resistance to the water flows both in favour of (or restricting) water entry and/or exit. Thus, membrane permeability not only to ions but also to water can contribute to plasticity together with the change in xylem tension as a consequence of the decrease in leaf water potential.

In our second hydraulic transition point (24 h treatment), the low *L*_pr_ values involved also an anatomical restriction enhancing the hydraulic resistance to water flows along the roots, suggesting an increment in the water flows through the cell-to-cell pathways (a more resistive pathway). The increase in suberization observed after 24-h treatment can be attributed to a completely different strategy. This is consistent with recent reports demonstrating that the cell-to-cell pathway might contribute significantly to the radial water uptake particularly during development (Knipfer and Fricke 2010; Knipfer *et al.* 2011; Gambetta *et al.* 2012, 2013; Caldeira *et al.* 2014; Suku *et al.* 2014). In wheat plants, a non-membranous pathway (apoplast) contributes to increase radial water uptake in the control but not in the NaCl-stressed plants (Fricke *et al.* 2013).

It is possible that the effectiveness of *B. vulgaris* to tolerate the saline stress could be associated with its capacity to maintain the expression level of the AQPs in the salt treatments (Fig. 4), as reported for other specific proteins strictly involved in salt tolerance (Chinnusamy *et al.* 2004, 2006). This statement cannot be made with certainty because aquaporin activity and protein expression were not tested here. The root strategy to maintain water flow is based on water and ion redistribution and adjusting the cell-to-cell pathway by means of its selected membrane permeability (Steudle 2000). A solely osmotic stress (PEG solute) shuts down the transcripts of the *Bv*PIP characterized in <4 h of treatment, which might contribute by increasing the root cell resistance to the water pathway. On the contrary, the cell-to-cell pathway in salt-treated *B. vulgaris* plants might contribute by increasing the capability to regulate water transfers because water permeability can be tuned to limiting (or maximizing) the resistance in concert with ion redistribution.

We could experimentally dissociate *L*_pr_ from *g*_s_ employing two strategies: (i) different cations—Na^+^ versus K^+^—to promote the stress and (ii) analysing *L*_pr_ enhancement when the salt treatment is interrupted (Fig. 5). Most of the studies in the literature are based on the analysis of *L*_pr_ decrease by means of an imposed stress condition or the presence of aquaporin inhibitors (e.g. Ehlert *et al.* 2009; Vandeleur *et al.* 2009). To our knowledge, this is the first work that explored altogether *L*_pr_ decrease and enhancement, as most of the works show *L*_pr_ inhibition and not its recovery. Whereas K^+^ and Na^+^ have distinct redistribution profiles, different *L*_pr_ recovery pathways for water are expected to be involved even in the presence of an equivalent change in the driving force along the SPAC for both situations. After stress treatments, the recovery of both hydraulic parameters (*L*_pr_ and *g*_s_) denoted two different strategies (Fig. 6). The enhancement in *L*_pr_ shows a profile (Figs 5C and 6) that is linked to ion redistribution (Na^+^ versus K^+^) and this is part of the root plasticity to prevent water loss. In the first transition point (4 h of salt treatment), the cation dependence of the *L*_pr_ profile highlights the participation of membrane permeability in root plasticity together with the change in xylem tension. On the other hand, *L*_pr_ recovery profiles observed after 24 h of salt treatment suggest that under our experimental conditions, root resistance to water flow does not differ between the ion source of the stress (Na^+^ or K^+^). This is consistent with an increase in the total root resistance and the observed strong endodermis suberization in both salt treatments (Fig. 3E and F). The recovery profile of NaCl treatments shows a coupled temporal dependence strategy where *g*_s_ and *L*_pr_ increase at the same rate. Both parameters increase at a slower pace when the plants were treated for 24 h compared with 4 h. Conversely, *g*_s_ and *L*_pr_ enhancement are clearly uncoupled in the KCl treatments (Fig. 6). The root shows the capacity to restore the water transport capacity before the water is transpired through stomata. It is well described in the literature that under salt stress, Na^+^ is redistributed to avoid toxicity, while K^+^ functions as an interchangeable ion all along the vasculature (particularly phloem) (Peng *et al.* 2004; Munns and Tester 2008; Karley and White 2009; Shabala *et al.* 2010; Flowers and Colmer 2015). In this context, the potassium gradient might be crucial in the root–shoot hydraulic signalling (Gajdanowicz *et al.* 2011). The profiles observed in Fig. 6 are consistent with sustaining a ‘hydraulic’ adjustment in the presence of NaCl compared with a ‘tuned’ adjustment caused by the redistribution in the case of KCl, which is clearly reflected in *g*_s_ and *L*_pr_ changes.

The proposed initial two set points—4 and 24 h extension in the imposed salt treatment—were selected because of the triggered distinguishable phenotypes in *B. vulgaris*. At 4 h of an imposed 200 mM salt stress, plants have lost turgor and osmotic adjustment has not been completed. In this situation, the *L*_pr_ recovery profile suggests a much higher root tuning capacity to modulate the water dynamics that affects the whole-plant water loss avoidance strategy. At 24 h of 200 mM salt stress, plants are gaining turgor, and the *L*_pr_ recovery profile suggests that the root versatility is more restricted as tolerance has already been triggered.

## Conclusions

Tolerance involves limiting water movement by increasing the total plant hydraulic resistance. *Beta vulgaris* osmotic adjustment is sustained by tuning *L*_pr_ and *g*_s_. Our work presents a quantitative analysis of the coordinated link between *L*_pr_ and *g*_s_ when the ion and water redistribution strategy takes place. Even when the xylem tension and apoplast pathway mediate plant water flows, the cell-to-cell pathway contributes as a key component to the capacity to transport water per unit surface and driving force in the SPAC (nicely demonstrated in the enhancement of *L*_pr_ after halting KCl treatment). Future research should explore the molecular basis for the different strategies that plants use to regulate their water balance and identify the imposed threshold of the cell-to-cell pathways in terms of hydraulic resistance.

## Sources of Funding

This work was supported by the Agencia Nacional para la Promoción Científica y Técnica [Préstamo BID PICT11-2239 and PICT14-0744], Consejo de Investigaciones Científicas y Técnicas (CONICET) Proyecto de Investigación Plurianual (PIP12-14) and Universidad de Buenos Aires UBACyT14-17, all grants to G.A.

## Contributions by the Authors

V.V., J.B. and G.A. were involved in the study conception and design. V.V. and J.B. planned and performed experiments and analysed data. J.B., G.S. and N.D.A. additionally participated in the design and data acquisition of the qRT-PCR experiments. V.V. and G.A. were involved in the analysis and interpretation of the data, discussion and writing the manuscript. All authors had intellectual input into the project.

## Conflict of Interest Statement

None declared.

## Supporting Information

The following additional information is available in the online version of this article –

**Figure S1.** Different concentrations of salt treatments.

**Table S1.** Osmotic potential measured for the leaf sap.

**Graph S1.** Root hydraulic conductivity determination.

**Table S2.** Accession number of genes and sequences of primer pairs used for qRT-PCR.

**Graph S2.** Root hydraulic conductivity determination after halting salt treatment.

**Graph S3.** Plant transpiration rate and RGR of leaf area.

**Tables S3–S5.** Quantitative real-time polymerase chain reaction statistical analysis.

Additional Information
