# Supplementary material for: Root hydraulic conductivity and adjustments in stomatal conductance: hydraulic strategy in response to salt stress in a halotolerant species
Source: AoB Plants. 2015 Nov 24;7:plv136. doi: 10.1093/aobpla/plv136 (PMC4683980; doi:10.1093/aobpla/plv136)
Supplement: Additional Information [file supp_7_plv136_index.html]

Root hydraulic conductivity and adjustments in stomatal conductance: hydraulic strategy in response to salt stress in a halotolerant species — Root hydraulic conductivity and adjustments in stomatal conductance: hydraulic strategy in response to salt stress in a halotolerant species — Additional Information 

# Root hydraulic conductivity and adjustments in stomatal conductance: hydraulic strategy in response to salt stress in a halotolerant species

## Additional Information

Additional Information

- Additional Information - Docx file
