# Supplementary material for: Root hydraulic conductivity and adjustments in stomatal conductance: hydraulic strategy in response to salt stress in a halotolerant species
Source: AoB Plants. 2015 Nov 24;7:plv136. doi: 10.1093/aobpla/plv136 (PMC4683980; doi:10.1093/aobpla/plv136)
Supplement: Additional Information [file supp_plv136_plv136supp.docx]

**SUPPORTING INFORMATION**

**File 1. Different concentration of salt treatment**

Plants exposed to NaCl at different concentrations (50, 100, 250, 500 mM) were individually tracked at different time intervals and images were acquired with a digital camera. The photographs correspond to four representative seedlings per treatment after 48h exposure to control or the indicated salt-concentration. Three replicates were performed per treatment, seven independent experiments were analyzed.

**
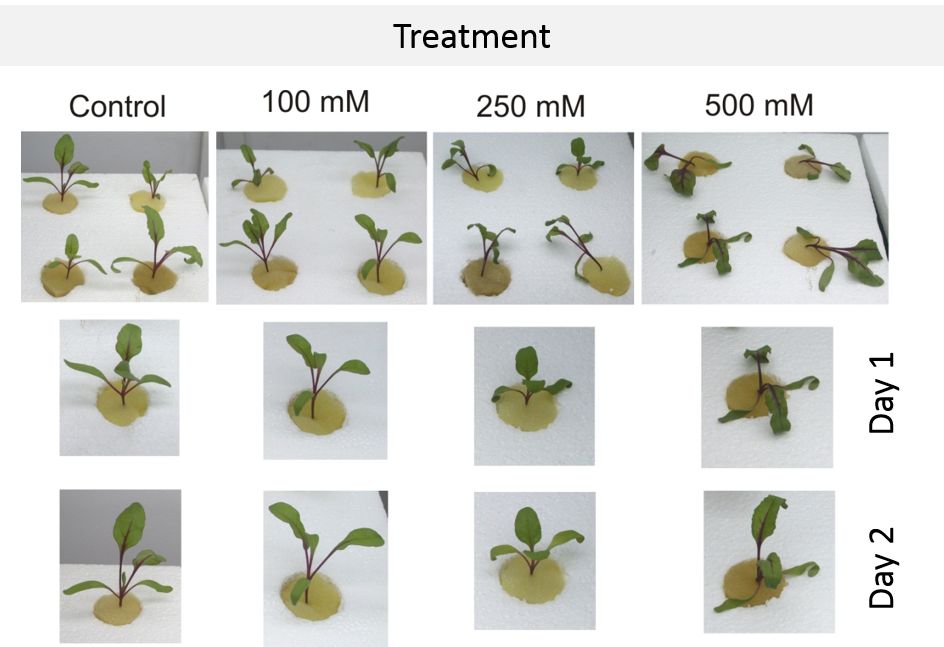
**

**File 2.**

**Table S1: Osmotic potential (*ψ_osm_*) measured for the leaf sap.** Values are leaf sap osmolality (MPa) expressed as mean values ± s.e. of 3 independent experiments (n:3). Grey cells represent statistically differences from control values (P < 0.05; Bonferroni test).

| Time (h) | 0 | 4 | 8 | 24 | 48 |
| --- | --- | --- | --- | --- | --- |
| Control | -0.717 ± 0.012 | -0.717 ± 0.015 | -0.754 ± 0.015 | -0.744 ± 0.012 | -0.667 ± 0.026 |
| NaCl | -0.715 ± 0.014 | -0.745 ± 0.022 | -0.809 ± 0.026 | -0.904 ± 0.098 | **-1.178 ± 0.078** |
| KCl | -0.722 ± 0.008 | -0.802 ± 0.015 | -0.831 ± 0.041 | **-0.950 ± 0.067** | **-1.174 ± 0.084** |

**File 3. *L_pr_* determination**

The plot shows the *J_v_: f(P)* relationship that allows the calculation of *L_pr_*. The data are a representative experiment where *J*_v_ was measured from one detopped root at three pressures (0.2, 0.3, 0.4 MPa). Three to five independent experiments were performed with similar results. In the equation, *J_v_*: water flux; *σ:* reflection coefficient of the membrane; *ΔP*, hydrostatic pressure, *Δπ*: osmotic pressure, *DW*: dry weight.

**
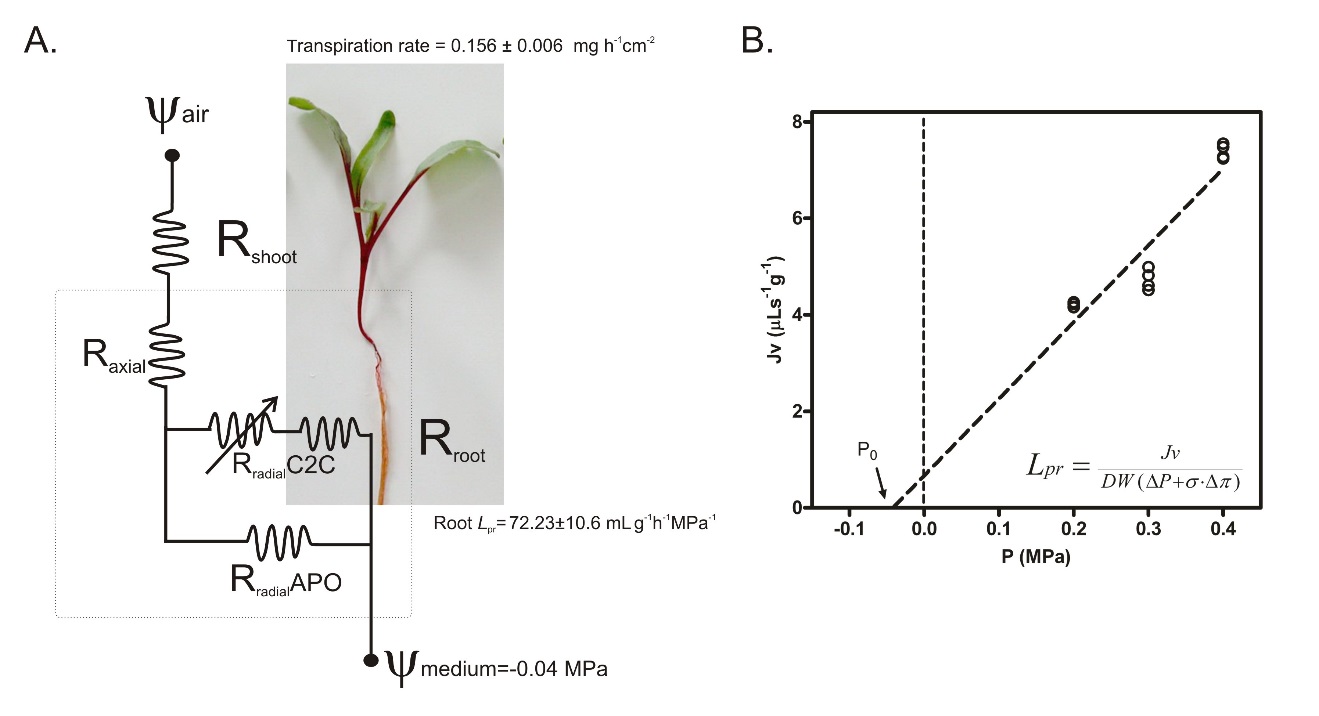
**

**File 4.**

**Table S2: Accession numbers of genes and sequences of primer pairs used for qRT-PCR.**

| Gene | Accession No | Sequence |
| --- | --- | --- |
| ***Bv*PIP1;1** | **GQ227845** | **5’-CGCCAAGAGAAGTGCTAGGGA-3’ (forward)**  **5’-GGGTTGATACCAGTGCCAGTAATG-3’ (reverse)** |
| ***Bv*PIP2;1** | **U60148** | **5’-CCAGCACCATTGTTTGACATGGGA-3’ (forward)**  **5’-ACCAACAGTAGCACAAGGGTCAGT-3’ (reverse)** |
| ***Bv*PIP2;2** | **GQ227846** | **5’-TTGTCTACTGCACTGCCGGAATCT-3’ (forward)**  **5’-TGGCACCAAGACATTGAGCTACCA-3’ (reverse)** |
| ***Bv*GAPDH** | **EF408234** | **5’-TCCACTGGTGTCTTCACCGACAAA-3’ (forward)**  **5’-CGTGCTCGTTGACACCAACAACAA-3’ (reverse)** |
| ***Bv*UBIep** | **FG345259** | **5’-AAGGAGTGCCCTAATGCTGAGTGT-3’ (forward)**  **5’-TGCTTTAATCGCCTCCAGCCTTCT-3’ (reverse)** |

**File 5. *L_pr_* determination after halting salt-treatment**

The *L_pr_* values are shown for plants exposed to 200 mM NaCl or 200 mM KCl treatment during 4 or 24 h. Bars represent mean ± s.e, discriminating measurements performed not only in the salt-treated solution (black bars) but also in control solution (white bars). There were not differences between *L_pr_* values obtained in control solution if compared with the treatment solution (identical letter, P>0.05, t-test). Data are mean values of three independent experiments, three replicas per experiment.

**
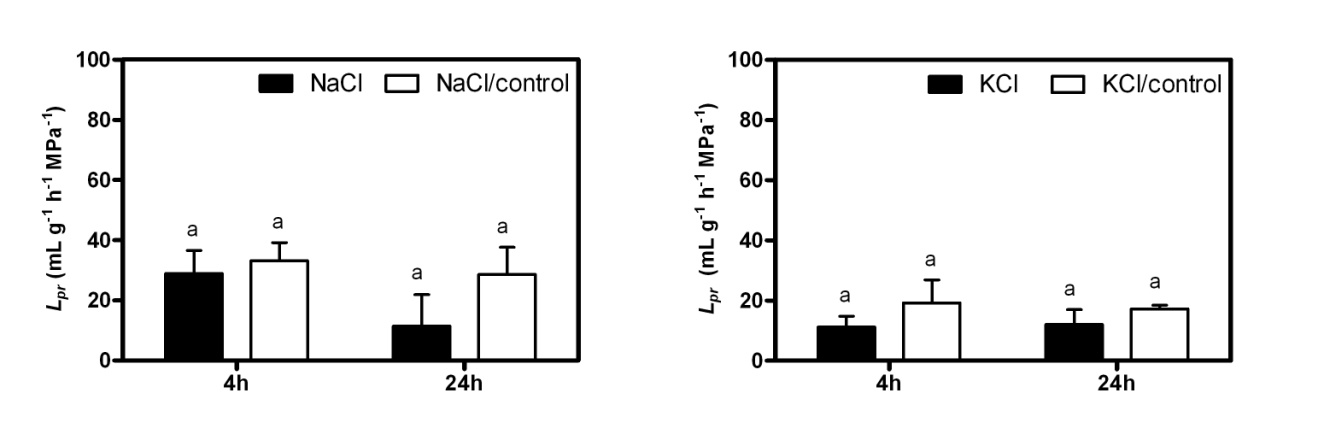
**

**File 6. Transpiration rate and relative growth rate of leaf area**

**A.** Average mass lost per hour per leaf area per day are shown in bars for each experimental condition (Control, 200 mM NaCl and 200 mM KCl). Data are mean values ± s.e., n =6-9 plants. Letters indicate treatments that are significantly different (P<0.001; Bonferroni test, n =2). **B.** The ratio of Ai (leaf area in a given time) and A_o_ (leaf area at the beginning of experiment) are plotted in function of time. The slope estimates the relative growth rate (RGR). **C.** Bars represent the RGR for each experimental condition. Data are mean values ± s.e., n =4. *** indicate treatments that are significantly different from zero (P<0.001).

**
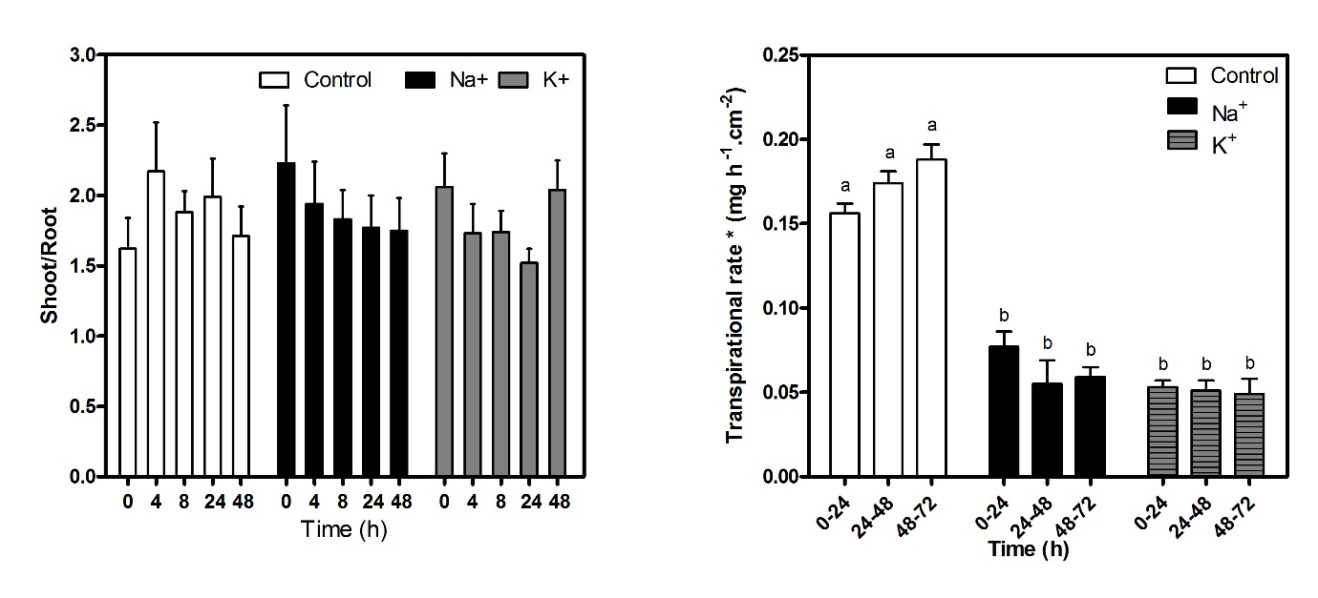
**

C.

B.

A.

**
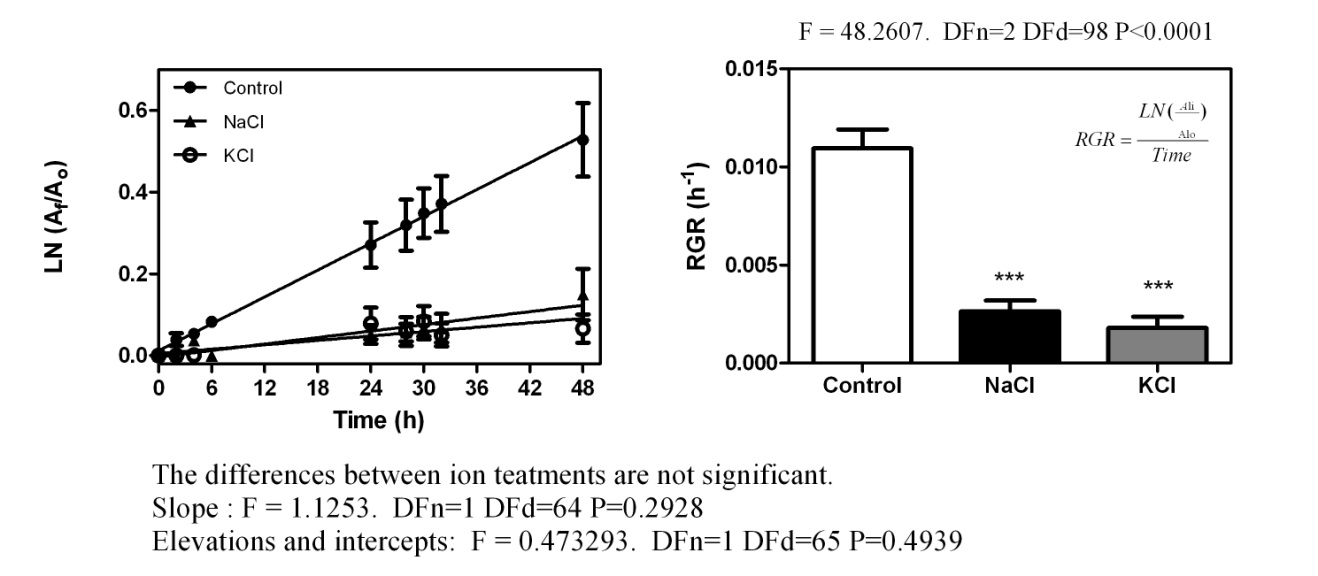
**

**File 7. qRT-PCR statistical analysis**

**Table S3: BvPIP2;1 qRT-PCR: statistical analysis**

Two-way ANOVA and Bonferroni post test p<0.05.

The interaction is considered not significant between TIME and TREATMENT (F = 1.03. DFn=8 DFd=30. The P value = 0.4340).

The TIME effect is considered very significant (attributable to circadian rhythms). TIME accounts for 30.25% of the total variance. F = 4.45. DFn=4 DFd=30. The P value = 0.0061.

The TREATMENT effect is considered not significant. F = 1.39. DFn=2 DFd=30. The P value = 0.2657.

|  | | Control | | | | | NaCl (200 mM) | | | | | KCl (200 mM) | | | | |
| --- | --- | --- | --- | --- | --- | --- | --- | --- | --- | --- | --- | --- | --- | --- | --- | --- |
|  |  | 0h | 4h | 8h | 24h | 48h | 0h | 4h | 8h | 24h | 48h | 0h | 4h | 8h | 24h | 48h |
| Control | 0h |  |  | * |  |  |  |  |  |  |  |  |  |  |  |  |
|  | 4h |  |  |  |  |  |  |  |  |  |  |  |  |  |  |  |
|  | 8h | * |  |  |  |  |  |  |  |  |  |  |  |  |  |  |
|  | 24h |  |  |  |  |  |  |  |  |  |  |  |  |  |  |  |
|  | 48h |  |  |  |  |  |  |  |  |  |  |  |  |  |  |  |
| NaCl (200 mM) | 0h |  |  |  |  |  |  |  |  |  |  |  |  |  |  |  |
|  | 4h |  |  |  |  |  |  |  |  |  |  |  |  |  |  |  |
|  | 8h |  |  |  |  |  |  |  |  |  |  |  |  |  |  |  |
|  | 24h |  |  |  |  |  |  |  |  |  |  |  |  |  |  |  |
|  | 48h |  |  |  |  |  |  |  |  |  |  |  |  |  |  |  |
| KCl (200 mM) | 0h |  |  |  |  |  |  |  |  |  |  |  |  |  |  | * |
|  | 4h |  |  |  |  |  |  |  |  |  |  |  |  |  |  |  |
|  | 8h |  |  |  |  |  |  |  |  |  |  |  |  |  |  |  |
|  | 24h |  |  |  |  |  |  |  |  |  |  |  |  |  |  |  |
|  | 48h | * |  |  |  |  |  |  |  |  |  |  |  |  |  |  |

**Table S4: BvPIP2;2 qRT-PCR: statistical analysis**

Two -way ANOVA and Bonferroni post test *p<0.05; ** p<0.01;*** p<0.001.

The interaction is considered significant between TIME and TREATMENT. Interaction accounts for 15.72% of the total variance. F = 3.13. DFn=8 DFd=30. The P value = 0.0108.

The TIME effect is considered extremely significant. Time accounts for 46.03% of the total variance. F = 18.32. DFn=4 DFd=30. The P value is < 0.0001.

The TREATMENT effect is considered extremely significant. TREATMENT accounts for 19.40% of the total variance. F = 15.44. DFn=2 DFd=30. The P value is < 0.0001.

|  | | Control | | | | | NaCl (200 mM) | | | | | KCl (200 mM) | | | | |
| --- | --- | --- | --- | --- | --- | --- | --- | --- | --- | --- | --- | --- | --- | --- | --- | --- |
|  |  | 0h | 4h | 8h | 24h | 48h | 0h | 4h | 8h | 24h | 48h | 0h | 4h | 8h | 24h | 48h |
| Control | 0h |  |  |  |  |  |  |  |  |  |  |  |  |  |  |  |
|  | 4h |  |  |  |  |  |  |  |  |  |  |  |  |  |  |  |
|  | 8h |  |  |  |  |  |  |  |  |  |  |  |  |  |  |  |
|  | 24h |  |  |  |  |  |  |  |  |  |  |  |  |  | ** |  |
|  | 48h |  |  |  |  |  |  |  |  |  | ** |  |  |  |  | ** |
| NaCl (200 mM) | 0h |  |  |  |  |  |  |  | * | * | *** |  |  |  |  |  |
|  | 4h |  |  |  |  |  |  |  |  |  | *** |  | * |  |  |  |
|  | 8h |  |  |  |  |  | * |  |  |  | ** |  |  |  |  |  |
|  | 24h |  |  |  |  |  | * |  |  |  | ** |  |  |  | * |  |
|  | 48h |  |  |  |  | ** | *** | *** | ** | ** |  |  |  |  |  |  |
| KCl (200 mM) | 0h |  |  |  |  |  |  |  |  |  |  |  | ** | ** | *** | *** |
|  | 4h |  |  |  |  |  |  | * |  |  |  | ** |  |  |  | * |
|  | 8h |  |  |  |  |  |  |  |  |  |  | ** |  |  |  |  |
|  | 24h |  |  |  | ** |  |  |  |  | * |  | *** |  |  |  |  |
|  | 48h |  |  |  |  | ** |  |  |  |  |  | *** | * |  |  |  |

**Table S5: BvPIP1;1 qRT-PCR: statistical analysis**

Two-way ANOVA and Bonferroni post test * p<0.05; ** p<0.01.

The interaction is considered very significant between TIME and TREATMENT. F = 3.96. DFn=8 DFd=30. The P value = 0.0027.

The TIME effect is considered not significant. TIME accounts for 11.72% of the total variance. F = 2.50. DFn=4 DFd=30. The P value = 0.0636.

The TREATMENT effect is considered very significant. F = 6.82. DFn=2 DFd=30. The P value = 0.0036.

|  | | Control | | | | | NaCl (200 mM) | | | | | KCl (200 mM) | | | | |
| --- | --- | --- | --- | --- | --- | --- | --- | --- | --- | --- | --- | --- | --- | --- | --- | --- |
|  |  | 0h | 4h | 8h | 24h | 48h | 0h | 4h | 8h | 24h | 48h | 0h | 4h | 8h | 24h | 48h |
| Control | 0h |  |  |  |  |  |  |  |  |  |  |  |  |  |  |  |
|  | 4h |  |  |  |  | * |  |  |  |  |  |  |  |  |  |  |
|  | 8h |  |  |  |  |  |  |  |  |  |  |  |  |  |  |  |
|  | 24h |  |  |  |  |  |  |  |  |  |  |  |  |  | * |  |
|  | 48h |  | * |  |  |  |  |  |  |  | ** |  |  |  |  | ** |
| NaCl (200 mM) | 0h |  |  |  |  |  |  |  |  |  |  |  |  |  |  |  |
|  | 4h |  |  |  |  |  |  |  |  |  | ** |  |  |  |  |  |
|  | 8h |  |  |  |  |  |  |  |  |  | ** |  |  |  |  |  |
|  | 24h |  |  |  |  |  |  |  |  |  | ** |  |  |  | ** |  |
|  | 48h |  |  |  |  | ** |  | ** | ** | ** |  |  |  |  |  |  |
| KCl (200 mM) | 0h |  |  |  |  |  |  |  |  |  |  |  |  |  |  |  |
|  | 4h |  |  |  |  |  |  |  |  |  |  |  |  |  |  |  |
|  | 8h |  |  |  |  |  |  |  |  |  |  |  |  |  |  |  |
|  | 24h |  |  |  | * |  |  |  |  | ** |  |  |  |  |  |  |
|  | 48h |  |  |  |  | ** |  |  |  |  |  |  |  |  |  |  |
